# Sol-Gel-Derived Hydroxyapatite-Carbon Nanotube/Titania Coatings on Titanium Substrates

**DOI:** 10.3390/ijms13045242

**Published:** 2012-04-24

**Authors:** Xiaoli Ji, Weiwei Lou, Qi Wang, Jianfeng Ma, Haihong Xu, Qing Bai, Chuantong Liu, Jinsong Liu

**Affiliations:** 1 Department of Prosthodontics, School and Hospital of Stomatology, Wenzhou Medical College, Wenzhou 325027, China; E-Mails: jixiaoli214@163.com (X.J.); louweiwei1125@163.com (W.L.); dentistmacn@yahoo.cn (J.M.); zhejiao1985@163.com (H.X.); qingqing_8429@126.com (Q.B.); dentliu@qq.com (C.L.); 2 State Key Laboratory of Oral Diseases, West China Stomatology Hospital, Sichuan University, Chengdu 610041, China; E-Mail: wqinno_875@163.com

**Keywords:** hydroxyapatite, carbon nanotubes, titania, anodization, sol-gel process

## Abstract

In this paper, hydroxyapatite-carbon nanotube/titania (HA-CNT/TiO_2_) double layer coatings were successfully developed on titanium (Ti) substrates intended for biomedical applications. A TiO_2_ coating was firstly developed by anodization to improve bonding between HA and Ti, and then the layer of HA and CNTs was coated on the surface by the sol-gel process to improve the biocompatibility and mechanical properties of Ti. The surfaces of double layer coatings were uniform and crack-free with a thickness of about 7 μm. The bonding strength of the HA-CNT/TiO_2_ coating was higher than that of the pure HA and HA-CNT coatings. Additionally, *in vitro* cell experiments showed that CNTs promoted the adhesion of preosteoblasts on the HA-CNT/TiO_2_ double layer coatings. These unique surfaces combined with the osteoconductive properties of HA exhibited the excellent mechanical properties of CNTs. Therefore, the developed HA-CNT/TiO_2_ coatings on Ti substrates might be a promising material for bone replacement.

## 1. Introduction

Titanium (Ti) has been widely used to fabricate biomedical materials because it has excellent biocompatibility, corrosion resistance, and mechanical properties [1,2]. However, the bio-inertness of the metallic surfaces inhibits the growth of bone tissue [3]. On the other hand, hydroxyapatite (HA; Ca_10_(PO_4_)_6_(OH)_2_) was widely used in hard tissue engineering such as bone and dentin formation, owing to its excellent osteoconductivity and biocompatibility [4]. However, the brittle nature and low fracture toughness of HA prevented its clinical application under load-bearing conditions. In order to overcome this shortage, HA has been applied as a coating on metallic surface, which combined the high mechanical strength of the metal with the excellent biocompatibility and bioactivity of the ceramic and is therefore suitable for implants in high load-bearing applications.

Carbon nanotubes (CNTs) exhibited outstanding mechanical, structural, thermal, chemical and optical properties [5–7], which attracted attention as reinforcement for high strength composites. CNTs have been added to HA to obtain nanocomposite coatings that combine the mechanical and biological properties of the individual materials and thus showed the improved mechanical performance [8,9]. Moreover, CNTs dissipated the residual stress in HA coatings [10].

Numerous strategies have been proposed to prepare HA, with the sol-gel method being the most favored. To prepare HA coatings from sol-gels, the calcium and phosphorus precursors were mixed at a molecular level for ensuring chemical homogeneity. In addition, the method is technically simple, cost-effective, and requires lower temperatures than do traditional processing methods [11,12]. Ti and HA have different thermal expansion coefficients, which may hinder the bonding strength between them. To improve the bonding between HA and Ti, a titania (TiO_2_) coating is formed on the Ti surface. Various methods have been adopted to form a TiO_2_ layer on the Ti substrate, such as anodization, thermal oxidation and the sol-gel process. Recently, anodization has been applied to obtain a rough, porous TiO_2_ layer on the surface of Ti [13,14]. This method is attractive because the TiO_2_ layer can be controlled by adjusting the processing conditions.

In this study, we intended to develop a novel method for fabricating HA-CNT/TiO_2_ double layer coatings on Ti substrates. Firstly, we developed a TiO_2_ by anodization. Then, the HA-CNT composite coating was coated on top of the TiO_2_ layer by the sol-gel process. The crystallization of the HA phase and the microstructure of the double layer coatings were investigated. The bonding strength of the coatings and their *in vitro* biological properties were also evaluated.

## 2. Results and Discussion

### 2.1. TiO_2_ Coating Phase and Morphology

**Figure 1:**
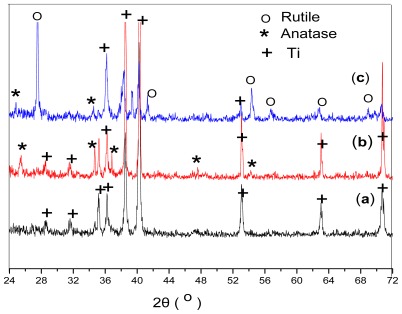
X-ray diffraction (XRD) spectra of the TiO_2_ layers before heat treatment (a) and after heat treatment at various temperatures for 1 h in air: (b) 450 °C; (c) 700 °C.

Heat treatment plays a vital role in the synthesis of TiO_2_ coatings, since it affects their morphology, crystallinity, and porosity and induces phase transformations. The crystalline forms of the TiO_2_ layer on the Ti substrate were analyzed before and after heat treatment at two temperatures (Figure 1). Before heat treatment, the XRD spectra of the TiO_2_ layer showed only reflections corresponding to the Ti substrate. Neither rutile nor anatase Ti crystalline phases were observed, indicating that the anodized film was amorphous TiO_2_. In order to remove water from the product, the layer should be calcined to a high temperature (above 450 °C). Heat treatment at 450 °C afforded an anatase phase, while treatment at 700 °C yielded a rutile phase. Hu *et al.* [15] have reported that TiO_2_ normally undergone an anatase-to-rutile phase transition at around 600–700 °C.

**Figure 2:**
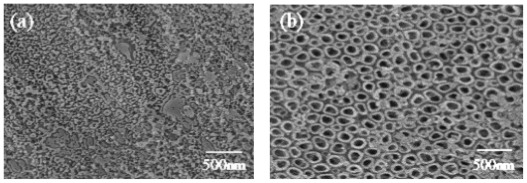

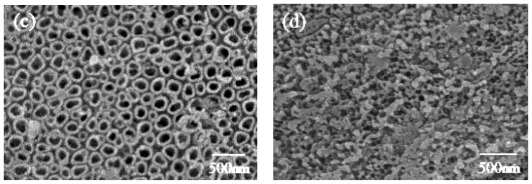
Field-emission gun scanning electron microscopy (FEG-SEM) images of the TiO_2_ surface structures obtained by anodizing the Ti substrates, at different voltages before heat treatment: (**a**) 5 V; (**b**) 10 V; (**c**) 20 V; (**d**) 30 V.

FEG-SEM observations revealed that the TiO_2_ structure probably formed pores or nanotubes, depending on the anodization voltage (Figure 2). At 5 V, TiO_2_ formed only a rough surface (Figure 2a). With increasing voltage, the TiO_2_ layer showed a more three-dimensional structure that included numerous open pores (Figure 2b,c) [16]. It was found that the pores were uniform and arrange regularly, and their diameters varied from 50 to 200 nm when the voltage was changed. At the highest applied voltage, the surface became cracked and irregular (Figure 2d), which was in agreement with the results reported by Ishizawa *et al*. [17]. Compared with the untreated Ti substrate, the anodized substrates showed rougher surface morphologies and a greater number of pores.

### 2.2. Double Layer Coating Phase and Morphology

**Figure 3:**
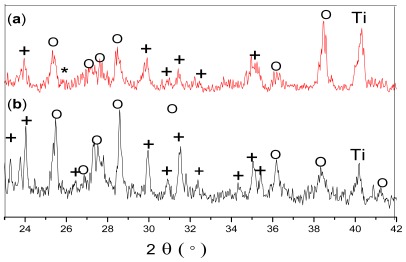
XRD spectra of the double layer coatings on Ti substrates after heat treatment at 550 °C for 30 min in air: (a) HA-CNT/TiO_2_ double layer coating; (b) HA/TiO_2_ double layer coating. (**O**): TiO_2_; (**+**): HA; (*): CNT.

**Figure 4:**
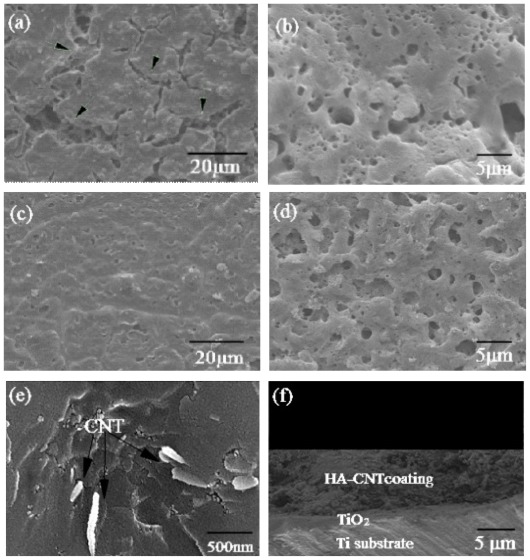
SEM images of the two coating systems on Ti substrate: (**a**) HA coating; (**b**) HA coating at high magnification; (**c**) HA-CNT/TiO_2_ double layer coating; (**d**) HA-CNT/TiO_2_ at high magnification; (**e**) micrograph of the fracture surface of the HA-CNT/TiO_2_ double layer coating; (**f**) HA-CNT/TiO_2_ double layer coating cross-sectional view.

Figure 3 shows the XRD patterns of the HA-CNT/TiO_2_(Figure 3a) and HA/TiO_2_(Figure 3b) double layer coatings on Ti substrates. No extraneous peaks appeared under the heat treatment, only peaks for HA, CNTs, TiO_2_ and Ti were detected. Weak and broadened peaks in the spectra might be due to the small grains of the coatings. There was a branched double peak at 2θ = 26°. Because the peak (26°) of CNTs overlapped with the peak (25.8°) of HA, the peak was weak and hardly confirmed CNTs in the double layer coatings. However, the existence of CNTs can be confirmed by SEM. The CNTs, as indicated by arrows in Figure 4e, were distributed in the HA matrix. These results suggested that the homogeneous dispersion of CNTs resulted in a higher rate of hydroxyapatite crystallization. The result was in agreement with the report by Najafi *et al*. [18], which showed that the synthesis of HA in the presence of CNTs had the best result in terms of homogenization of the carbon nanotube dispersion and faster crystallization of hydroxyapatite.

**Figure 5:**
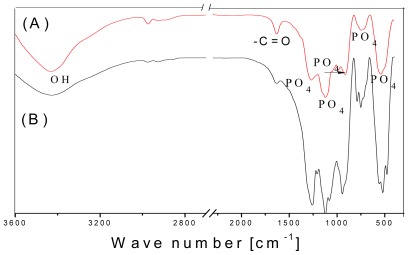
Fourier transform infrared spectroscopy (FTIR) spectra of the double layer coatings on Ti substrates after heat treatment at 550 °C for 30 min in air: (A) HA-CNT/TiO_2_ double layer coating; (B) HA/TiO_2_ double layer coating.

FTIR spectroscopy confirmed some features of the coatings that could not be observed by XRD analysis. The expected orthophosphate (PO_4_^3−^) and hydroxyl (–OH) peaks were clearly presented in the spectra of both HA coatings (Figure 5). The PO_4_^3−^ ions in the apatite structure gave a sharp and broad IR absorbance between 800 and 1100 cm^−1^. PO_4_^3−^ stretching and bending modes were seen at 557 cm^−1^, 944 cm^−1^, and 1121 cm^−1^. A broad peak at around 3000–3600 cm^−1^, centered at 3428 cm^−1^, could be attributed to OH stretching. Suchanek *et al.* have reported an OH vibration peak at around 630 cm^−1^ for HA, but this peak was not evident in our spectra [19]. Interestingly, a C=O stretching peak attributed to the carbonyl on the CNTs was visible at 1630 cm^−1^ (Figure 5A).

With the SEM observation, the morphologies of the HA and HA-CNT/TiO_2_ double layer coatings on the Ti substrate were slightly different. The HA-CNT/TiO_2_ double layer coatings appeared to be highly dense and uniform, while the simple HA coating had numerous microcracks (Figure 4a,c). The discrepancy in the thermal expansion coefficients between HA (15.2 × 10^−6^ °C^−1^) and Ti (8.6 × 10^−6^ °C^−1^) probably resulted in excessive strain during the heating and cooling processes. In our method, the mismatch of thermal expansion coefficients between them was reduced by TiO_2_ layer (9.0 × 10^−6^ °C^−1^). The anodized coatings helped in reducing the microcracks, also yielding a denser coating [20]. Moreover, CNTs further reduced the crack propagation because of their function in transferring and eliminating residual stress in the HA/CNTs composite coatings, which had been proved by Lin *et al* [21]. The HA and HA-CNT/TiO_2_ double layer coatings had similar rough surface microstructures at high magnification (Figure 4b,d). The rough surface of the HA coatings has been reported to be advantageous for cell attachment and proliferation [22].

The cross sectional surface of coatings showed that the coatings with a uniform thickness of about 7 μm were very compact and dense (Figure 4f). No pores or microcracks were detected in the coatings. The layer bonded firmly to the substrate and held a uniform thickness throughout the Ti surface. Moreover, there were no delaminations or cracks at the interlayer/topcoat interface, suggesting that both Ti substrate/TiO_2_ interlayer and TiO_2_ interlayer/HA-CNT topcoat interfaces had very good bonding capabilities.

### 2.3. Bonding Properties

**Figure 6:**
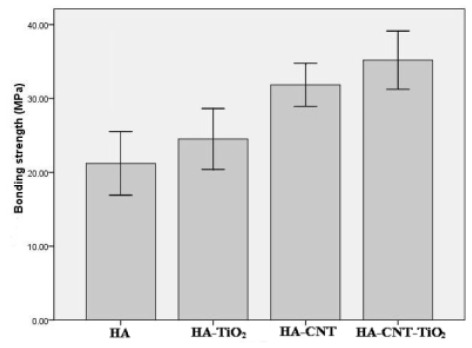
Bonding strengths of different coatings deposited on Ti substrates.

To evaluate the mechanical properties of the double layer coatings, the bonding strength of the different coatings was measured, and the results presented in Figure 6. As expected, the insertion of the TiO_2_ layer and CNTs significantly improved the bonding strength of the layer to the Ti substrate. The HA-CNT/TiO_2_ double layer coatings bound more strongly than the HA, HA/TiO_2_ and HA-CNT coatings (*p* < 0.05). The bonding strength of the HA-CNT/TiO_2_ double layer coatings increased to as high as 35.2 MPa, which constituted an approximately 65.9% enhancement with respect to that of the HA single coating (21.2 MPa) and was much higher than that of electrophoretic deposition HA coatings [9]. The HA-CNT layer bonded more tightly to the TiO_2_ layer than to the bare Ti substrate. Because the mismatch of the thermal expansion coefficients of HA and Ti was alleviated by the addition of TiO_2_, resulting in the decreased residual stress in the coatings. These results proved that the oxidation of Ti substrate improved the attachment of HA to metal substrates, which was in agreement with the findings of Hautaniemi *et al.* [23]. The CNTs also helped prevent the HA-CNT coating from peeling off by acting as a reinforcement network [24].

**Figure 7:**
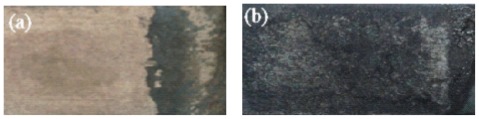
Morphology of failed surface of (**a**) HA-CNT and (**b**) HA-CNT/TiO_2_ showing the coatings peeled off from the substrate after adhesion test.

The images of the failure surfaces of HA-CNT/TiO_2_ and HA-CNT double layer coatings after adhesion strength tests were shown in Figure 7. Failure occurred entirely at the Ti substrate/coating interface for the HA-CNT coating (Figure 7a), While some debris of the coatings remained on the surface of HA-CNT/TiO_2_ double layer coatings, which showed failure occurred between interlayer and topcoat (Figure 7b). Based on these results, it was confirmed that the bonding strength of the coating layer was dictated by the coating defects and the substrate type.

### 2.4. *In Vitro* Cellular Assessment

**Figure 8:**
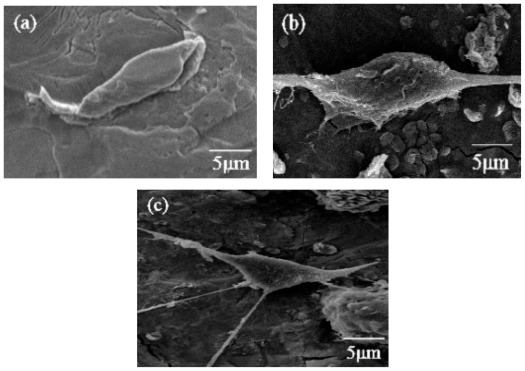
SEM images of preosteoblast MC3T3-E1 cells growing on each sample after 24 h of incubation: (**a**) bare Ti; (**b**) HA coating; and (**c**) HA-CNT/TiO_2_ double layer coating. TiO_2_ was heat-treated at 450 °C for 1 h.

In order to evaluate cell attachment to the coatings, preosteoblast MC3T3-E1 cells were fixed after 24 h of culturing with the different coatings and imaged (Figure 8). As is shown in Figure 8a, the cells did not spread out well on the bare Ti control. By contrast, the cells on the HA coating gradually adhered to and spread out on the substrates (Figure 8b). Furthermore, the cells on the HA-CNT/TiO_2_ double layer spread out further and had more filopods than those on the bare Ti and the HA/TiO_2_ coating (Figure 8c). The anodized substrates, which have rougher surface morphologies and numerous micron-sized pores, could improve protein adhesion. Moreover, the porous edges were beneficial for cell adhesion [25]. Recent studies have demonstrated that osteoblast cells adhered to the surface of functionalized MWCNTs. Zanello *et al.* tried to control cell growth on CNTs by functionalizing them and demonstrated that neutrally charged CNTs sustained osteoblast growth and bone formation [26]. These results indicate that during the initial incubation, the anodized Ti and CNTs in the double layer coating promoted preosteoblast cell attachment and adhesion.

**Figure 9:**
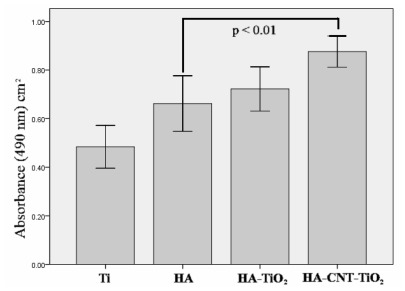
Proliferation of MC3T3-E1 cells cultured on each sample for 5 d. Bare Ti substrate was used as a control.

To assay proliferation on the different coatings, preosteoblast MC3T3-E1 cells were cultured on each material for 5 d. Cell proliferation was enhanced on each HA coating when compared to that on bare Ti (Figure 9). All the coatings (HA, HA/TiO_2_, HA-CNT, and HA-CNT/TiO_2_) were assayed, and the HA-CNT/TiO_2_ double layer coating showed a significantly higher cell proliferation rate relative to the pure HA coating (*P* < 0.01). A study by George *et al*. tested the response of MWCNTs to human lung epithelial cells, osteoblast-like cells and primary osteoblast cells. They suggested that the dimensions and spacing of CNTs may be key to determining subsequent cell spreading and proliferation [27]. The findings in this research also suggest that adding CNTs to HA coatings enhance its bioactive properties.

## 3. Experimental Section

### 3.1. Preparation of Ti Substrates

Ti sheets (TA_2_, Baoji Non-ferrous Metals Co., China) measuring 10 × 10 × 2 mm were used as the substrates. Their surfaces were polished with silicon carbide papers (grit #180, 320, 600, 800, and 1000), followed by ultrasonic cleaning in acetone, ethanol, and distilled water. Before the experiment, samples were etched in an acidic solution (HF/HNO_3_/H_2_O = 1:3:10) for 3 min to remove the oxide layer that naturally formed in air atmosphere.

### 3.2. Preparation of HA-CNT/TiO_2_ Coating

Samples were anodized at room temperature with a direct current power supply. A graphite plate and the Ti sample were used as the cathode and the anode, respectively. The substrates were anodized in HF/H_2_O with H_3_PO_4_ at 5–30 V for 1 h to form nanostructured Ti surfaces. The solution was stirred to homogenize the electrolyte and allow gasses to escape from the Ti surface. After anodization, the samples were cleaned with distilled water, dried, and then heat treated at 450 °C or 700 °C in air for 2 h. After heat treatment, the samples were ultrasonically cleaned in acetone, ethanol, and distilled water and finally dried to obtain the TiO_2_ film.

The HA sol was fabricated from its precursors, Ca(NO_3_)_2_·4H_2_O and P_2_O_5_. Stoichiometric amounts of Ca(NO_3_)_2_·4H_2_O and P_2_O_5_ were dissolved in separate ethanol solutions. Next, the P_2_O_5_ solution was slowly added to the Ca(NO_3_)_2_·4H_2_O solution to achieve a calcium (Ca)/phosphorus (P) ratio = 1.67. Then, 1 wt% carboxyl-multiwalled CNTs (MWCNTs) with a diameter of 10–20 nm and length of 0.5–2 μm (Chengdu Institute of Organic Chemistry, China) were added to the HA sol with vigorous stirring for 24 h; the resulting mixture was subsequently aged at room temperature for 48 h to obtain a clear sol. Carboxylated MWCNTs are easily dispersible in solvents.

Next, the anodized Ti substrates (anodized in HF/H_2_O with H_3_PO_4_ at 20 V for 1 h and heat treated at 450 °C) were slowly dipped into the composite sols at a withdrawing rate of 5 cm/min. As a comparison, the Ti substrates and the anodized Ti substrates were dipped into the HA sols. The gels obtained from the sols were dried at 150 °C for 30 min and then heat-treated at 550 °C for 30 min in order to fix the double layer coatings. Controls with pure HA and HA-CNT (no TiO_2_) were also tested and characterized.

### 3.3. Characterization

The crystalline phases in the coatings were analyzed by X-ray diffraction (XRD; D8 Focus, Bruker Co., Germany) and Fourier transform infrared spectroscopy (FTIR; Equinox 55, Bruker Co., Germany). For FTIR analysis, the coating was scraped off from the treated surface, mixed with high-purity KBr powder, and compacted into pellet form. The surface and cross-sectional morphologies of the double layer coatings were studied by Field-emission gun scanning electron microscopy (FEG-SEM) (JSM-6700F, JEOL Co., Japan) and scanning electron microscopy (SEM; S3000N, Hitachi Co., Japan).

The bonding strength of each coating layer was measured by adhering the double layer coating to an uncoated Ti plate with epoxy resin cured at 100 °C for 1.5 h. After cooling, the Ti plate was pulled away at a loading rate of 1 mm/min until the coating layer failed, and the bonding strength was determined by dividing the maximum load by the surface area. Five identical specimens were tested for each data point, which is represented as mean ± SD (n = 5). Single factor analysis of variance (ANOVA) technique was used to assess the statistical significance of results between groups.

### 3.4. *In Vitro* Cellular Assessment

Specimens were sterilized in 120 °C steam for 1 h and then placed in 24-well plates. MC3T3-E1 preosteoblast cells, at a density of 1 × 10^4^ cells/mL, were plated on each specimen, and CP Ti (unalloyed commercially pure Ti) was used as a control. The cells were cultured in α-minimum essential medium (α-MEM; GIBCO, USA) supplemented with 10% fetal bovine serum (FBS; GIBCO, USA), 2 mM l-glutamine, and 100 U/mL penicillin at 37 °C in humidified 5% CO_2_. After 24 h of incubation, the cells were washed with phosphate-buffered saline (PBS) solution; then, the cells were detached using a trypsin-EDTA solution, and the living cells were counted using a haemocytometer (Superior Co., Germany). To observe the cell morphology, the specimens were fixed in 2.5% glutaraldehyde for 4 h at 4°C and dehydrated gradually in 70%, 80%, 90%, and 95% (v/v) ethanol solutions for 15 min each and then twice in absolute ethanol. For examination by SEM (S3000N, Hitachi Co., Japan), surfaces with immobilized cells were dehydrated by critical point drying and coated with gold.

Cell activity was determined by using 3-(4,5-dimethylthiazol-2-yl)-2,5-diphenyl tetrazolium bromide (MTT) assay, The cells (3 × 10^4^ cells/mL) were seeded on the coating specimens in 24-well plates containing α-MEM and placed for 5 days at 37 °C, then washed with PBS solution, 20 μL of MTT (5 mg/mL) was added to each well and incubated for 4 h at 37 °C. At the end of the assay, the blue formazan reaction product was dissolved by adding 100 μL dimethyl sulphoxide (DMSO) and transferred to a 96-well plate. The absorbance was determined at a wavelength of 490 nm using a microplate reader (Infinite^®^ M200 PRO, Tecan Group Ltd., Switzerland).

## 4. Conclusions

Uniform, crack-free HA-CNT/TiO_2_ double layer coatings were successfully fabricated on Ti substrates, which a TiO_2_ interlayer obtained by means of anodization and a HA-CNT composite topcoat deposited by sol-gel process. The HA-CNT/TiO_2_ double layer coatings had the highest bonding strength, showing resistance up to 35.2 MPa, which were much higher than that of electrophoretic deposition HA coatings. The insertion of the TiO_2_ layer and CNTs in the coatings promoted preosteoblast cell adhesion and proliferation. These findings suggest that adding CNTs to HA coatings affords promising materials for bone replacement. Furthermore, this study suggests that a TiO_2_ bonding coat, introduced via anodization, may be useful to improve the adhesion of HA and HA-TiO_2_ coatings to titanium substrates.
